# Bone marrow from periacetabular osteotomies as a novel source for human mesenchymal stromal cells

**DOI:** 10.1186/s13287-023-03552-9

**Published:** 2023-11-03

**Authors:** Maximilian Handke, Anastasia Rakow, Debora Singer, Lea Miebach, Frank Schulze, Sander Bekeschus, Janosch Schoon, Georgi I. Wassilew

**Affiliations:** 1 Center for Orthopaedics, Trauma Surgery and Rehabilitation Medicine, University Medicine Greifswald, 17475 Greifswald, Germany; 2 ZIK Plasmatis, Leibniz Institute for Plasma Science and Technology (INP), Felix- Hausdorff-Str. 2, 17489 Greifswald, Germany

**Keywords:** Bone marrow, Mesenchymal stromal cells, Mononuclear cells, Periacetabular osteotomy, Regenerative medicine, Total hip arthroplasty

## Abstract

**Background:**

Bone marrow-derived mesenchymal stromal cells (BM-MSCs) are used in regenerative medicine and related research involving immunomodulatory, anti-inflammatory, anti-fibrotic and regenerative functions. Isolation of BM-MSCs from samples obtained during total hip arthroplasty (THA) is routinely possible. Advanced age and comorbidities of the majority of patients undergoing THA limit their applicability. Our study aimed to evaluate the potential of bone marrow obtained during periacetabular osteotomy (PAO) as a novel source of BM-MSCs from young donors by analyzing cell yield and cell characteristics.

**Methods:**

Bone samples were obtained from the anterior Os ilium or superior Os pubis during PAO and from the femoral cavity during primary THA. Isolation of bone marrow-derived mononuclear cells (BM-MNCs) was performed by density gradient centrifugation. The samples from PAO and THA patients were compared in terms of BM-MSC yield, colony formation and the proportion of BM-MSCs within the BM-MNC population using flow cytometry analysis. The cells were characterized based on the expression of BM-MSC-specific surface markers. The functionality of the cells was compared by quantifying post-thaw viability, metabolic activity, proliferation capacity, senescence-associated beta galactosidase (SA-β-gal) expression, trilineage differentiation potential and major secretome proteins.

**Results:**

Isolation of BM-MNCs was possible in a reliable and reproducible manner when using bone from PAO containing more than 0.24 g bone marrow. PAO patients were younger than patients of the THA group. Bone obtained during PAO contained less bone marrow and led to a lower BM-MSC number after the first cell culture passage compared to BM-MSCs obtained during THA. BM-MSCs from PAO samples are characterized by a higher proliferation capacity. This results in a higher yield in cell culture passage two, when normalized to the sample weight. BM-MSCs from PAO patients showed increased secretion of TGF-β1, TIMP2, and VEGF upon osteogenic differentiation. BM-MSCs from PAO and THA patients revealed similar results regarding the onset of SA-β-gal expression and trilineage differentiation capacity.

**Conclusions:**

We suggest that bone obtained during PAO is a promising novel source for BM-MSCs from young donors. Limited absolute cell yield due to low sample weight must be considered in early cell culture passages and might be critical for the range of clinical applications possible for BM-MSCs from this source. The higher proliferation capacity and increased growth factor secretion of BM-MSCs from young donors may be beneficial for future regenerative cell therapies, in vitro models, and tissue engineering.

**Supplementary Information:**

The online version contains supplementary material available at 10.1186/s13287-023-03552-9.

## Background

Mesenchymal stromal cells (MSC) are one of the most frequently studied and used cell types in regenerative medicine [1, 2]. As defined by the International Society for Cellular Therapy (ISCT), human MSCs are characterized by their adherent growth on standard cell culture plastic, their expression of CD105, CD73 and CD90, the absence of expression of CD45, CD34, CD14, CD11b, CD79alpha, CD19 and HLA-DR, as well as by their ability to differentiate into the adipogenic, osteogenic and chondrogenic phenotype [3]. It is possible to stimulate endogenous MSCs for mobilization as well as to administer allogenic MSCs systemically in the course of cytotherapy [4]. The ability of self-renewal by asymmetric division and the multipotency of MSCs allow for versatile applications, including the repair of bone defects [5]. Multiple studies showed promising results using MSCs in the therapy of non-union fractures and bone defects [6–9]. Furthermore, MSC-based therapies are likely to be beneficial in the treatment of osteoarthritis, considering their paracrine function and potential for cartilage regeneration [10–12]. In the field of tissue engineering, a recent pre-clinical application of MSCs in cartilage discs was successful regarding the regeneration of critical size femoral bone defects [13]. Playing a key role in future therapies, there is a high demand for MSCs, especially from young, healthy donors [14, 15]. Isolation is mainly performed from bone marrow, as well as adipose and placental tissue [14]. It was shown that human MSCs from adipose and placental tissues secrete increased levels of tissue-factor compared to bone marrow-derived MSCs (BM-MSCs), which might be the cause of thrombosis and embolism when administered intravenously [16]. Tissue for the isolation of MSCs can either be obtained as a by-product during surgical interventions (bone marrow and adipose tissue) and after birth (placental tissue) or by procedures carried out only for the purpose of cell isolation. A well-established procedure for obtaining BM-MSCs is the iliac crest bone marrow aspiration [17]. Although being a painful procedure with a risk of complications, the yield of BM-MSCs is relatively low [18]. In addition, the isolation of BM-MSCs obtained from the distal femur, proximal tibia, humeral head, sternum, vertebral body, and the radius via bone puncture and aspiration was demonstrated successfully [19]. Gold standard in musculoskeletal research is the isolation of BM-MSCs from bone marrow of the femur, obtained during total hip arthroplasty (THA) [20]. Considering the average age of patients undergoing surgical interventions like THA, the question arises whether advanced age and comorbidities limit the use of the isolated cells [21–24]. At our internationally renowned center for diagnosis and treatment of hip dysplasia more than 150 periacetabular osteotomies (PAO) are performed annually, making our institution a European leader in the field of sophisticated pelvic surgery. Since PAO is applied to treat hip dysplasia in adolescents and young adults, patients are respectively young [25]. During the procedure, three osteotomies are performed to enable reorientation of the acetabulum and to thereby achieve an improved coverage of the femoral head [26]. Bone marrow-containing bone wedges are sometimes resected from the anterior Os ilium or superior Os pubis and discarded as surgical waste in the course of PAO [27]. So far, bone wedges from PAO patients have not been recognized as a potential cell source from young patients. Human BM-MSCs have been shown to be superior to fat-derived MSCs in an osteochondral in vivo model [13]. We see a high potential of PAO-derived samples for the isolation of BM-MSCs and their application in research and application in regenerative medicine after thorough characterization.

Thus, the aim of this study was to determine whether bone obtained during PAO could serve as a novel source of BM-MSCs. To this end, analyses of biological characteristics and functional properties were performed, comparing BM-MSCs obtained during PAO or THA. We hypothesize that BM-MSCs from patients undergoing PAO are superior to BM-MSCs from THA especially in the context of osteogenic potential. We follow the overall aim to establish a novel cell source for future regenerative cell therapies suitable for application in biomaterial assisted bone-forming cell therapies.

## Methods

### Patient recruitment

For this study, bone samples from 35 patients undergoing PAO to treat hip dysplasia were used. Bone from 11 patients undergoing primary THA due to osteoarthritis of the hip served as controls. Written informed consent for the collection and characterization of bone samples and corresponding cells as well as for the publication of anonymized patient data including age and sex was given by all patients. Baseline patient data and information on the use and characteristics of the individual bone material can be found in Additional file 1: Table S1. Patients with active malignancy, chronic infection, or immunosuppressive therapy were excluded from the study. Information on preoperative concomitant medication and comorbidities of the patients whose cells were analyzed regarding functionality are provided in Additional file 1: Table S2. The independent ethics committee (IEC) of the University Medicine Greifswald granted ethics approval (BB 087/21) in accordance with the World Medical Association Declaration of Helsinki.

### Harvesting of bone material

Bone wedges containing intertrabecular bone marrow were collected from the anterior Os ilium or superior Os pubis during PAO. The harvesting did not extend the routine procedure of PAO nor did it result in any additional risks for patients. In clinical practice, the bone wedges are discarded as surgical waste. During primary THA, the femoral marrow cavity is opened and prepared. In this process, trabecular bone containing intertrabecular bone marrow was collected. The specimens collected in the course of PAO and primary THA were used for the isolation of BM-MNCs and subsequent isolation of BM-MSCs.

### Isolation and cultivation of BM-MSCs

Immediately after intraoperative harvesting, the bone material was stored for a maximum of 4 h at 4 °C without addition of any buffer until further processing. Bone marrow was separated from the compacta using scalpel and forceps under sterile conditions. The collected bone marrow was weighed in a Petri dish. BM-MNCs were isolated via density gradient centrifugation as previously described [22, 28]. Isolated BM-MNCs were seeded on cell culture flasks with a cell density of 5 × 10^5^/cm^2^ and cultured at 37 °C, 95% humidity and 5% CO_2_ atmosphere. Expansion medium (EM), containing low glucose Dulbecco's Modified Eagle's Medium (DMEM, PAN Biotech) supplemented with 10% fetal bovine serum (FBS, Sigma-Aldrich), 100 U/ml penicillin (Gibco), 100 µg/ml streptomycin (Gibco) and 2 mM L-alanyl-L-glutamine (GlutaMAX, Gibco), was changed 48 h after seeding and subsequent media changes were performed twice a week. Cells were detached with 0.05% trypsin containing 0.02% EDTA (PAN-Biotech). Cell numbers and cell viability were quantified using the TC20 Automated Cell Counter (Bio-Rad Laboratories) and seeded at a density of 2,400 cells/cm^2^ on day (d) 14 of primary cell culture. BM-MSCs were cryopreserved in cell culture passage two in low glucose DMEM containing 12.5% human serum albumin (HSA, Biotests Pharma GmbH) and 10% dimethyl sulfoxide (AppliChem). By thawing BM-MSCs from cell culture passage two and expanding them under standard cell culture conditions until 80% confluence, a sufficient number of cells was obtained for the following experiments regarding cell viability, cell proliferation, osteogenic, adipogenic and chondrogenic differentiation. BM-MSCs from both groups were expanded under standardized and equal conditions in terms of in vitro time, cultivation protocols and freeze–thaw protocols.

### Histology

Bone samples were fixed with 4% formaldehyde (FA, Herbeta) in phosphate-buffered saline (PBS, Bio&Sell GmbH) for 24 h and stored at 4 °C in PBS containing 100 U/ml penicillin and 100 µg/ml streptomycin until further processing. Bone was decalcified with 20% ethylenediaminetetraacetic acid (Carl Roth) solution for multiple days. The bone was then rinsed with tap water, dehydrated with an ascending ethanol series and stored in xylene (Carl Roth) for 24 h, followed by embedding in paraffin/xylene (1:1). 7 µm slices were cut with a microtome (Leica RM2255). Staining with hematoxylin and eosin (both Carl Roth) was performed by the “progressive method” as frequently described [29].

### Colony-forming unit assay

Triplicates of isolated BM-MNCs were seeded on 6-well tissue culture plates with a cell density of 5 × 10^5^/cm^2^ (5 × 10^6^ cells per well) and cultured in 2 ml EM. 48 h after seeding, cells were rinsed twice with pre-warmed EM. A second media change was performed at d4. Cells were fixed at d7 with 4% FA for 10 min and subsequently stained by using the Blue Alkaline Phosphatase Substrate KIT III (Vector® Labs) according to the manufacturer’s protocol. Aggregates of > 20 alkaline phosphatase (ALP) positive cells were counted as a Colony-forming unit (CFU) using a phase contrast microscope.

### Flow cytometry analyses

Cell surface marker expression analyses by flow cytometry were performed to quantify the proportion of the BM-MSC population within the BM-MNC population immediately after BM-MNC isolation and to characterize the isolated BM-MSCs. For this purpose, cells were thawed and expanded until cell culture passage three. Cells were detached with accutase (PAN Biotech), washed twice with PBS and incubated with fluorescently-labeled monoclonal antibodies (all BioLegend) for 15 min at room temperature. Antibodies used (positive markers): CD105 (PerCP cyanine 5.5.), CD73 (brilliant violet 421) and CD90 (phycoerythrin). Antibodies used (DUMP markers): CD14, CD19, CD34, CD45 and HLA-DR (all APC cyanine 7). Following the 15 min of incubation, cells were washed, suspended in FACS buffer (BioLegend) and analyzed by flow cytometry (CytoFLEX LX, Beckman-Coulter). Data analysis was done using Kaluza 2.2 software (Beckman-Coulter).

### Cell viability and proliferation

BM-MSCs from cell culture passage three were seeded on 48-well tissue culture plates (6 wells / donor) with a density of 1.8 × 10^3^ cells per well in 200 µl EM. 24 h after seeding (referred to as d0), at d4 and d7, cell viability was determined by using a resazurin-based assay (PrestoBlue, Invitrogen) according to the assay manual. Medium changes were performed at d0 and d4. Following the quantification of fluorescence intensities, plates were washed with PBS and stored at  − 80 °C. Collected plates were thawed, and DNA quantification was performed (CyQuant assay, ThermoFisher) according to the assay manual. The fluorescence intensities of the CyQuant (485 nm excitation, 530 nm emission) and PrestoBlue (560 nm excitation, 590 nm emission) assays were quantified with a plate reader (TECAN Infinite M200 PRO). Population doublings were calculated by relating the fluorescence intensities of the CyQuant assay of d4 and d7 to the value of d0 using the following formula: log (value d4 or d7/value d0) / log (2). Post-thaw viability and recovery of BM-MNCs was determined immediately after thawing by automated cell counting. In addition, 4.8 × 10^3^ of thawed cells were seeded on 48-well tissue culture plates, cultured in 200 µl EM. 24 h after seeding, cell viability was determined by PrestoBlue and normalized to cell number (CyQuant).

### Detection of senescence-associated beta galactosidase

BM-MSCs from cell culture passage two were thawed and expanded in EM until cell culture passage four and five. After passaging by trypsinization, triplicates of 4.8 × 10^3^ cells were seeded on 48-well tissue culture plates, expanded in EM and fixed at d4 for 10 min in 4% FA and then stored at 4 °C in PBS. Beta galactosidase signals were detected with the CellEvent™ Senescence Green Detection Kit (ThermoFisher) according to the to the manufacturer’s protocol. In addition, nuclei staining using 1 µg/ml DAPI (Sigma-Aldrich) in dH2O was performed. Fluorescence imaging under constant settings was performed using a fluorescence microscope (Invitrogen EVOS FL). The mean beta galactosidase signal intensities of each image were quantified using ImageJ.

### Osteogenic differentiation

BM-MSCs from cell culture passage three were seeded on 48-Well tissue culture plates (6 wells / donor) with a cell density of 4.8 × 10^3^ cells per well and cultivated with 200 µl EM. 24 h after seeding (d0), the induction of osteogenic differentiation was performed by replacing EM with osteogenic medium (OM). Non-stimulated cells (cultivation in EM) served as negative controls of differentiation. OM consisted of low glucose DMEM supplemented with 10% FBS, 10 mM beta glycerolphosphate disodium salt, 50 µM L-Ascorbic acid 2-phosphate sesquimagnesium salt, 100 nM dexamethasone (all Sigma-Aldrich), 100 U/ml penicillin, 100 µg/ml streptomycin and 2 mM L-alanyl-L-glutamine (GlutaMAX). Medium changes were performed twice a week. To evaluate the osteogenic differentiation potential, cellular ALP activity and collagen I synthesis were quantified as early osteogenic markers and matrix mineralization was quantified as a late osteogenic marker following induction of osteogenic differentiation.

The ALP activity was quantified at d0 (24 h after seeding), d4, and d7 by para-Nitrophenylphosphate (pNPP, Sigma-Aldrich) conversion to 4-Nitrophenol (pNP) as previously described [30]. In brief, cells were washed with PBS and 250 µl AP-puffer containing 100 mM NaCl (Merck), 100 mM Tris(hydroxymethyl)aminomethane (Merck) and 1 mM MgCl_2_ (Carl Roth). 100 µl AP-puffer and 100 µl AP-substrate (1 mg/ml pNPP, 1 mol/l diethanolamine (Sigma-Aldrich) were added. After 10 min incubation at 37 °C, pNPP conversion was stopped by adding 200 µl NaOH 1 M (Sigma-Aldrich). The accumulation of pNP was then quantified by absorption at 405 nm with a plate reader (TECAN Infinite M200 Pro).

Cell culture supernatants from d7 of osteogenic differentiation were collected and stored at − 80 °C. After thawing the supernatants, the human procollagen type I N-terminal propeptide (P1NP) concentrations were quantified with an ELISA-based assay (Elabscience) according to the manual.

Matrix mineralization was quantified at d14 and d21. Cells were washed with PBS, fixed with 4% FA solution and stained with 0.5% Alizarin Red S (Sigma-Aldrich) solution. Using a phase contrast microscope (ECHO Rebel), the matrix content was documented. Colorimetric quantification of calcium matrix dissolved by cetylpyridinium chloride (Sigma-Aldrich) was performed by absorption measurement at 562 nm as previously described [31].

### Adipogenic differentiation

BM-MSCs from cell culture passage three were seeded on 48-well tissue culture plates (six wells/donor) with a cell density of 9.0 × 10^3^ cells per well and cultivated with 200 µl EM. After 24 h of cultivation (d0), adipogenic differentiation was induced by replacing EM with adipogenic medium. Adipogenic medium was based on DMEM 4500 mg/l glucose (PAN Biotech), supplemented with 10% FBS, 100 U/ml penicillin, 100 µg/ml streptomycin, 2 mM L-alanyl-L-glutamine (GlutaMAX), 1 µM dexamethasone, 2 µM insulin from bovine pancreas (Th. Geyer), 500 µM 3-isobutyl-1-methylxanthine (Sigma-Aldrich) and 100 µM indomethacin (Sigma-Aldrich). Non-stimulated cells (cultivation in EM) served as negative controls of differentiation. Medium changes were performed twice a week. On d10 and d14, cells were washed with PBS, fixed with 4% FA solution and stained with 1 µg/ml DAPI in dH_2_O solution. Using a TECAN plate reader (Infinite M200 PRO), the fluorescence intensity was measured (355 nm excitation, 460 nm emission) in order to determine the relative cell number. Subsequently, cells were stained with 0.1% NileRed (Sigma-Aldrich) in PBS solution, and the fluorescence was determined again (485 nm excitation, 538 nm emission). For normalization, NileRed fluorescence intensities were related to the respective DAPI fluorescence intensities. Fluorescence imaging of fat droplets was realized using a fluorescence microscope (Invitrogen EVOS FL).

### Chondrogenic differentiation

After trypsinization, 3 × 10^5^ cells from cell culture passage four were centrifuged at 400 × g for 10 min in 15 ml falcon tubes. EM was replaced by 500 µl chondrogenic medium based on DMEM 4500 mg/l glucose supplemented with 173 µM L-Ascorbic acid 2-phosphate sesquimagnesium salt, 0.1 µM dexamethasone, 0.35 mM L-proline (Sigma-Aldrich), 1 mM sodium pyruvate (PAN Biotech), 1.25 mg/ml HSA (Biotest AG), 6.25 µg/ml insulin–transferrin–sodium selenite media supplement (Sigma-Aldrich), 19.1 µM linoleic acid (Sigma-Aldrich), 100 U/ml penicillin, 100 µg/ml streptomycin and 2 mM L-alanyl-L-glutamine (GlutaMAX). In order to induce chondrogenic differentiation, 10 ng/ml mammalian-derived recombinant human TGF-β1 (BioLegend) was added. Falcon tubes were centrifuged at 400 × g for 10 min. For the next 21 days, cell spheroids were cultivated under 37 °C and 5% CO_2_ atmosphere with medium changes twice a week. To evaluate the chondrogenic differentiation potential, the size of the spheroids, histological staining of glycosaminoglycans by alcian blue, the total protein content of the chondrogenic spheroids and the proteoglycan content of the spheroids were quantified. Chondrogenic spheroids were washed with PBS and cryopreserved at − 80 °C until quantification of total protein and proteoglycan content.

Proteoglycan quantification was performed by detecting sulfated glycosaminoglycans with a dimethylmethylene blue (DMMB) assay [32] and quantification of absorbance at 516 nm. Total protein content of the lysates was quantified by a Bradford protein assay (Pierce Coomassie Protein Assay Kit, ThermoFisher) according to the assay manual.

For histology, pellets were fixed with 4% FA, dehydrated with an ascending ethanol series, and embedded in paraffin via xylene. 7 µm slices were cut with a microtome prior to alcian blue/nuclear fast red staining. In brief, sections were deparaffinized with xylene, rehydrated with a descending ethanol series, placed in 3% acetic solution for 10 min, stained with 1% alcian blue 8GX (Morphisto) solution in 3% acetic acid (Th. Geyer) for 30 min, counterstained with nuclear fast red (Carl Roth) for 5 min and dehydrated with an ascending ethanol series. To compare the size of the chondrogenic spheroids, the edge of each spheroid in a phase contrast microscope image (ECHO Rebel) was traced, and area quantification of cross sections was performed using ImageJ.

### Multiplex assay

In order to analyze growth factors and cytokines secreted by the BM-MSCs, a LEGENDplex customized human 12-plex assay kit (BioLegend) was used according to the manufacturer's instructions. At d7 of cell culture in EM or after osteogenic stimulus with OM, medium supernatants from six patients of each group were pooled from six wells each and stored at -80 °C until analysis. Media supernatants were collected at d7 of osteogenic differentiation since at this time point BM-MNCs acquire an osteogenic phenotype, are characterized by high metabolic activity and produce osteogenic matrix. Analyzed soluble factors were hepatocyte growth factor (HGF), interleukin-1 receptor antagonist (IL-1RA), interleukin-6 (IL-6), interleukin-8 (IL-8), macrophage colony-stimulating factor (M-CSF), osteoprotegerin (OPG), stem cell factor (SCF), monocyte chemoattractant protein-1 (MCP-1), transforming growth factor-beta 1 (TGF-β1), stromal cell-derived factor (SDF), tissue inhibitor of metalloproteinases 2 (TIMP2), and vascular endothelial growth factor (VEGF).

### Statistical analysis

For exploratory statistical analysis and descriptive data plotting, GraphPad Prism 9.4.1 was used. All samples and data were included in the analyses, and all data points are shown as individual values. Shapiro–Wilk test was performed to test for normal distribution. If normal distribution was confirmed, F-test followed to compare variances. In case of significantly different variances, an unpaired *t*-test with Welch’s correction was performed. Otherwise, an unpaired t-test was used. For non-normally distributed data sets, Mann–Whitney testing was performed. The significance level was set to *p* < 0.05. Plots with normally distributed data sets are shown with mean value (bar) ± SD (standard deviation). Medians with IQR (interquartile range) were plotted when datasets were non-normally distributed.

## Results

### Cell yield and cell characterization

In order to evaluate whether bone obtained during PAO can be a novel source for BM-MSCs, the cell yield was evaluated by cell number determination of isolated BM-MNCs and BM-MSCs and subsequent surface marker expression analysis.

**Fig. 1:**
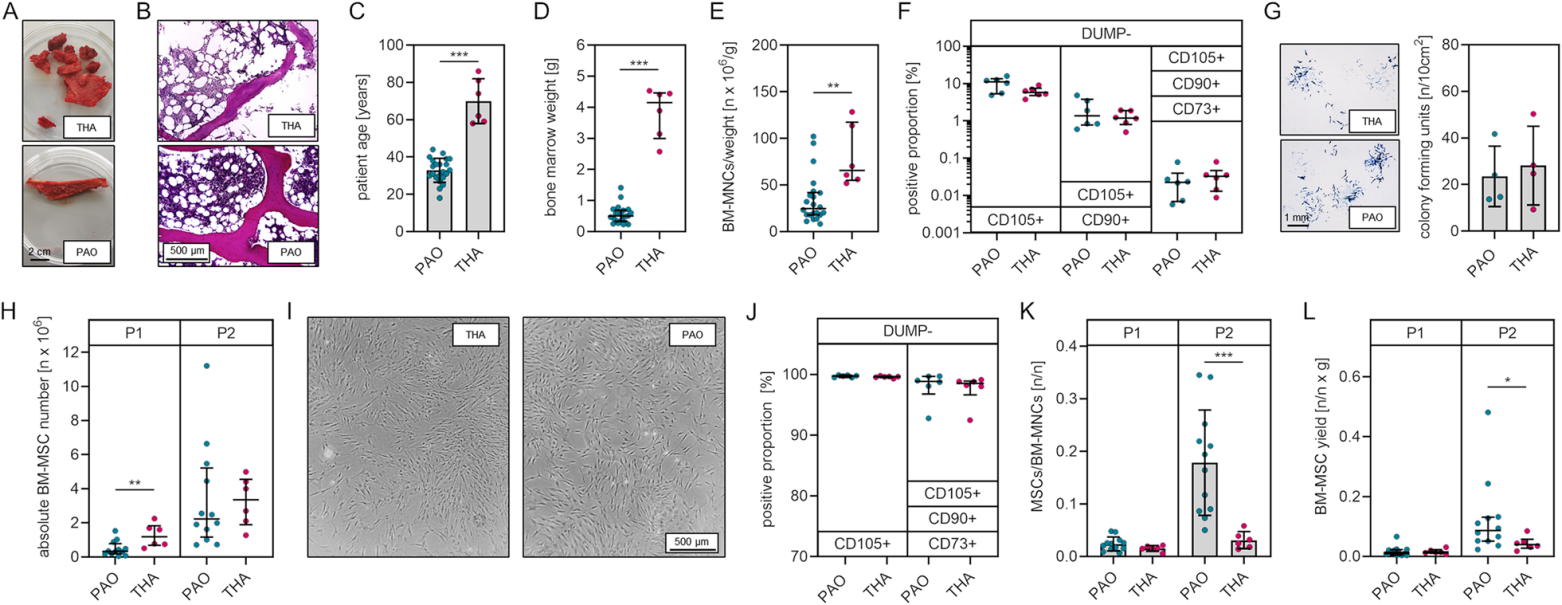
Isolation of BM-MNCs and subsequent isolation of BM-MSCs is possible using bone harvested during PAO. **A** Representative bone sample harvested during THA, a representative bone wedge harvested during PAO. **B** Representative hematoxylin and eosin staining of cancellous bone obtained during PAO and THA. **C** Age of enrolled patients. **D** Weight of harvested bone marrow. **E** Number of BM-MNCs isolated normalized to bone marrow weight. **F** MSC marker expression analyses of BM-MNCs. **G** Representative bright filed images of colony-forming units positive for alkaline phosphatase (left) and numbers of counted colonies (right). **H** Absolute numbers of isolated BM-MSCs after cell culture passage 1 (P1) and cell culture passage 2 (P2). **I** Phase contrast microscopic images of BM-MSCs on day 14 of primary cell culture. **J** MSC marker expression analyses of isolated BM-MSCs. **K** BM-MSC number relative to the number of BM-MNCs seeded after cell culture passage 1 and cell culture passage 2. **L** Weight corrected BM-MSC yield after cell culture passage 1 and cell culture passage 2 [normally distributed data: mean ± SD; non-normally distributed data: median with IQR; levels of significance: *p < 0.05, **p < 0.01, ***p < 0.001]

The bone samples obtained during PAO had a wedge shape consisting of compact and trabecular bone, while the bone samples obtained during THA consisted of trabecular bone only (Fig. 1A). Hematoxylin and Eosin staining of trabecular bone samples revealed the presence of intertrabecular bone marrow in samples from both groups (Fig. 1B). A minimum of 0.24 g bone marrow was required to reliably isolate BM-MNCs (Additional file 1: Table S1). In four cases, the bone samples contained less than this minimum amount of bone marrow, resulting in unsuccessful isolations. PAO patients were significantly younger (*p* ≤ 0.001) than THA patients (mean age [years] ± SD: PAO, 32.9 ± 6.4; THA, 70.0 ± 12.0) (Fig. 1C). Bone samples obtained during PAO contained significantly less bone marrow (p ≤ 0.001) compared to material obtained during THA (median weight [g] with (IQR): PAO, 0.50 (0.35); THA, 4.15 (1.47)) (Fig. 1D). The number of BM-MNCs in relation to the bone marrow weight was found to be significantly lower (*p* = 0.001) in the PAO group compared to the THA group (median number of cells/bone marrow mass [n × 10^6^/g] with (IQR): PAO, 24.7 (24.1); THA, 65.5 (62.3)) (Fig. 1E). Flow cytometry analysis of the BM-MNCs showed no significant differences between the two groups regarding the proportions of CD105 + cells (endothelial cells, macrophages, monocytes, BM-MNCs, p = 0.057), CD105 + / CD90 + cells (endothelial cells, BM-MNCs, p = 0.314) and CD105 + / CD90 + / CD73 + cells (BM-MNCs, p = 0.310) (Fig. 1F). The quantification of the number CFUs formed after seeding BM-MNCs did not show significant differences between the two groups (Fig. 1G). The quantified absolute cell numbers indicate that the BM-MSC yield is significantly lower (*p* = 0.010) after the first cell culture passage following isolation from PAO samples if compared to cell numbers following isolation from THA samples (median cell numbers [n × 10^6^] with (IQR): PAO, 0.33 (0.59); THA, 1.19 (1.15)). However, there was no significant difference (*p* = 0.617) of the absolute cell numbers following isolation of BM-MSCs from samples of both groups after cell culture passage two, indicating a comparable and sufficient BM-MSC yield (Fig. 1H). The isolated and expanded BM-MSCs from both groups showed typical spindle-shaped morphology (Fig. 1I). Flow cytometry analysis of isolated BM-MSCs of each group at cell culture passage three revealed no significant difference (*p* = 0.485) of proportions of cells positive for MSC specific surface markers (CD105, CD90, CD73) between BM-MSCs isolated from samples obtained during PAO and THA (Fig. 1J). Normalization of the BM-MSC number after cell culture passage one (d14) to the number of BM-MNCs initially seeded showed no significant differences (*p* = 0.157) between the two groups, whereas quantification of the BM-MSC number after cell culture passage two revealed a significantly higher number (*p* ≤ 0.001) of BM-MSCs from PAO samples when considering the number of initially seeded BM-MNCs (mean normalized cell number [n/n] ± SD: PAO, 0.178 ± 0.100; THA, 0.031 ± 0.016) (Fig. 1K). Calculation of the weight corrected cell yield (number of BM-MSCs/number of BM-MNCs x BM weight) showed no significant differences (*p* = 0.598) between the two groups at cell culture passage one and a significantly higher weight corrected cell yield (*p* = 0.025) of BM-MSCs from PAO samples (median weight corrected cell yield [n/n × g] with (IQR): PAO, 0.087 (0.080); THA, 0.040 (0.030) (Fig. 1L).

Taken together, BM-MSC isolation from bone obtained during PAO is feasible. The absolute numbers of BM-MSC isolated from bone obtained during PAO at cell culture passage two are comparable to the numbers of BM-MSCs isolated from bone obtained during THA. The weight corrected cell yield at cell culture passage two of BM-MSCs from PAO samples is higher if compared to that of BM-MSC from THA samples.

### Cellular behavior assessment

In order to compare basic cell properties, post-thaw viability and recovery, proliferation capacity and metabolic activity were quantified. In addition, cells were stained for senescence-associated beta galactosidase (SA-β-gal).

**Fig. 2:**
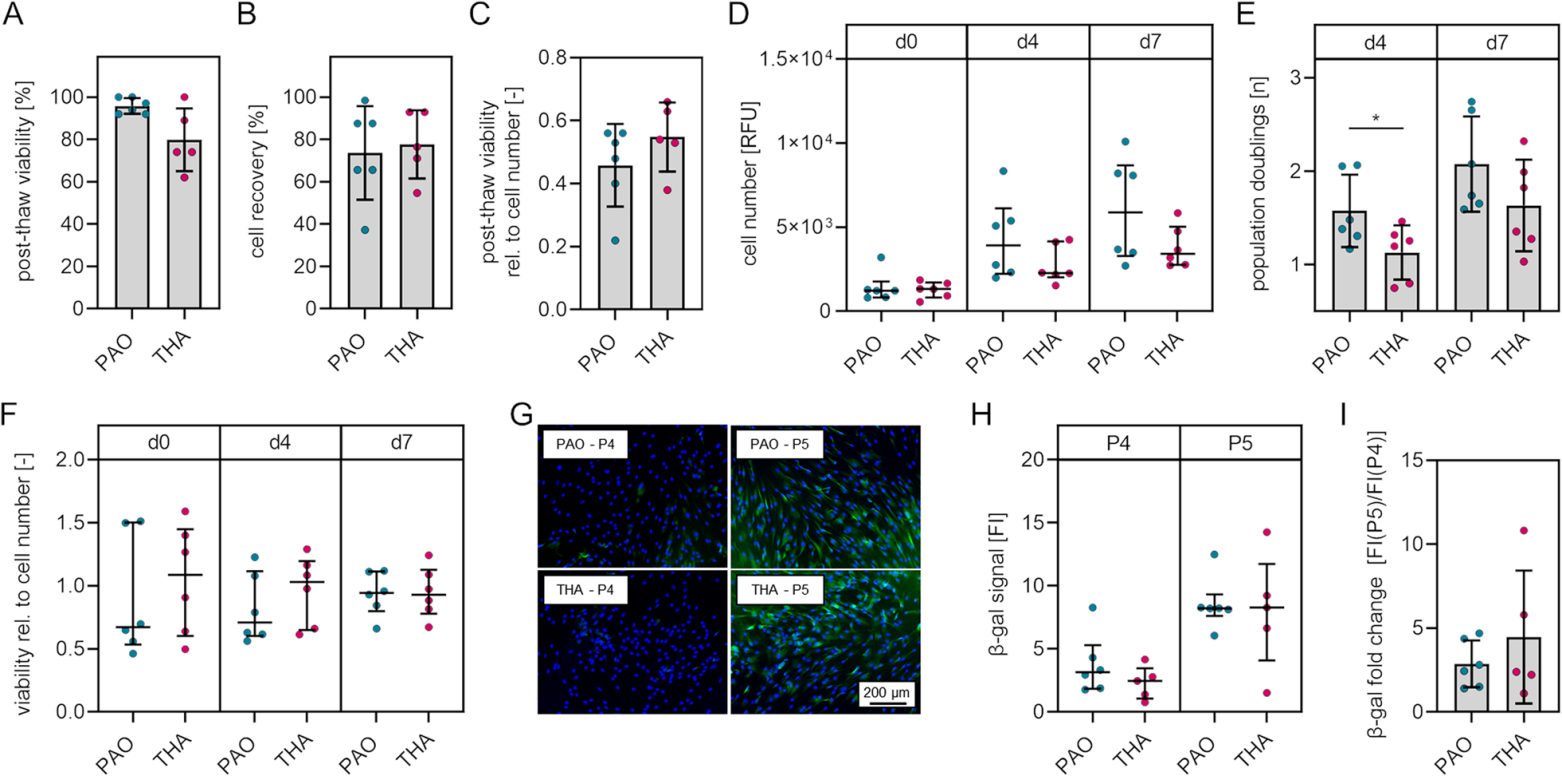
Post-thaw viability, proliferation capacity, metabolic activity and senescence of BM-MSCs isolated from bone samples obtained during PAO and THA. **A** Cell viability after thawing of cells. **B** Cell recovery after thawing. **C** Metabolic activity of thawed cells 24 h after seeding relative to cell number. **D** Cell numbers determined by DNA quantification at days 0 (24 h after seeding), 4 and 7. **E** Population doublings of cells at days 4 and 7. **F** Metabolic activity relative to cell number at days 0 (24 h after seeding), 4 and 7. **G** Representative fluorescence images of BM-MSCs from cell culture passage 4 and 5 stained for senescence-associated beta galactosidase. Green, beta galactosidase; blue, nuclei. **H** Intensities of the beta galactosidase signals. **I** Fold change of the beta galactosidase signals. [normally distributed data: mean ± SD; non-normally distributed data: median with IQR; levels of significance: *p < 0.05] Abbreviation: β-gal, beta galactosidase

Automated cell counting immediately after thawing of cells from cell culture passage two revealed no significant differences between BM-MSCs from PAO samples and BM-MSCs from THA samples regarding the proportion of viable cells (*p* = 0.072) (Fig. 2A) and cell recovery (*p* = 0.746) by means of the ratio of the cell number after thawing and the cell number before freezing (Fig. 2B). Quantification of metabolic activity normalized to the cell number 24 h after seeding of thawed cells as a marker of the post-thaw viability also revealed no significant difference (*p* = 0.458) between the two groups (Fig. 2C). DNA quantification in cell culture passage three revealed no significant differences of cell numbers at d0 (24 h after seeding, *p* = 0.589), d4 (*p* = 0.180) and d7 (*p* = 0.310) (Fig. 2D). Calculation of the population doublings revealed a significantly higher (*p* = 0.048) proliferation rate at cell culture d4 (mean population doublings [n] ± SD: PAO, 1.58 ± 0.39; THA, 1.13 ± 0.29) whereas this effect was not statistically significant (p = 0.156) at cell culture d7 (Fig. 2E). The normalized cellular metabolic activity in cell culture passage three was not significantly different between BM-MSCs from both groups at d0 (24 h after seeding, *p* = 0.699), d4 (p = 0.394) and d7 (*p *≥ 0.999) (Fig. 2F). Fluorescence imaging indicated the onset of SA-β-gal expression in cell culture passage five of either BM-MNCs isolated from PAO samples or THA samples (Fig. 2G). Fluorescence imaging and subsequent quantification of fluorescence intensities revealed that BM-MSCs from one out of six PAO samples showed a specific beta galactosidase signal at cell culture passage four and that BM-MSCs from one out of five THA samples do not express beta galactosidase at cell culture passage five (Fig. 2H, Additional file 1: Fig. S1). The signal intensities were not significantly different between BM-MSCs from both groups at cell culture passage four (*p* = 0.247) and cell culture passage five (*p* = 0.792) (Fig. 2H). Calculation of the fold increase of signal intensities did also not reveal significant differences between the two groups (*p* = 0.433) (Fig. 2I).

Taken together, BM-MSCs isolated from bone obtained during PAO and from bone obtained during THA are not differently affected by freezing/thawing, are not different in metabolic activity and the onset of SA-β-gal expression. BM-MSCs from PAO samples are characterized by a higher proliferation capacity compared to BM-MSCs from THA samples.

### Trilineage differentiation potential of BM-MSCs

Osteogenic, adipogenic, and chondrogenic differentiation assays were performed to evaluate trilineage differentiation, a key feature of BM-MSCs’ functionality.

**Fig. 3:**
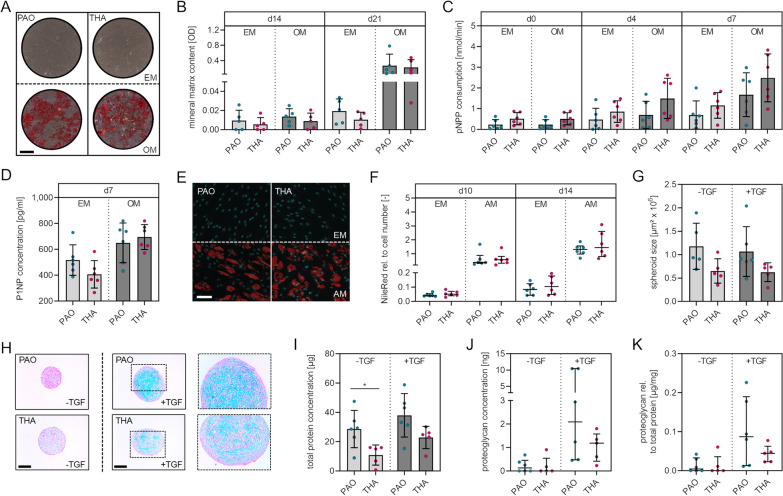
Multilineage differentiation potential of BM-MSCs isolated from bone samples obtained during PAO and THA. **A** Representative images of Alizarin Red stained cells following 21 days of culture in expansion medium (EM) and osteogenic medium (OM). Scale bar, 500 µm. **B** Quantification of the mineral matrix content following Alizarin Red staining at day 14 and 21 of osteogenic differentiation. **C** Quantification of pNPP consumption indicating ALP activity at day 0 (24 h after osteogenic stimulus), 4 and 7. **D** P1NP concentration as a marker for collagen I synthesis in cell culture supernatants collected at day 7 of osteogenic differentiation. **E** Representative fluorescence microscopy of BM-MSCs after staining with NileRed (red, fat droplets) and DAPI (cyan, nuclei) at day 14 of culture in EM and adipogenic medium (AM). Scale bar, 100 µm **F** Quantification of fat droplets normalized to cell number following NileRed staining at day 10 and 14 of osteogenic differentiation. **G** Quantification of the spheroids’ surface areas following 21 days of 3D culture without chondrogenic stimulus (−TGF) and with chondrogenic stimulus (+TGF). **H** Representative images of alcian blue stained sections of spheroids. Scale bar, 250 µm. **I** Total protein content of spheroids at day 21. **J** Total proteoglycan content of spheroids at day 21. (K) Total proteoglycan content normalized to total protein content of spheroids. [normally distributed data: mean ± SD; non-normally distributed data: median with IQR; levels of significance: *p < 0.05]

Mineral content of the osteogenic matrix was not significantly different at d14 (*p* = 0.388) and d21 (*p* = 0.785) of osteogenic differentiation of BM-MSCs obtained from PAO in comparison to BM-MSCs obtained from THA (Fig. 3A, B). ALP activity was also not significantly different between the two groups at d0 (EM, *p* = 0.101; OM, *p* = 0.099), d4 (EM, *p* = 0.237; OM, *p* = 0.127) and d7 (EM, *p* = 0.249; OM, *p* = 0.234), either following culture in EM or following osteogenic stimulus with OM (Fig. 3C). Moreover, no significant difference in P1NP accumulation in the cell culture supernatant of BM-MSCs obtained from PAO in comparison to BM-MSCs obtained from THA either with (*p* = 0.577) or without (*p* = 0.0119) osteogenic stimulus was observed (Fig. 3D). The quantity of fat droplets as a marker for adipogenic differentiation potential was not significantly different at d10 (*p* = 0.937) and d14 (*p* = 0.818) of adipogenic differentiation between the two groups (Fig. 3E, F). Quantification of chondrogenic spheroid sizes revealed a trend toward bigger spheroids based on BM-MSCs from PAO in comparison to spheroids based on BM-MSCs from THA (mean spheroid size [µm^2^] ± SD: −TGF-β1 PAO, 1.18 ± 0.49; −TGF-β1 THA, 1.18 ± 0.49; + TGF-β1 PAO, 1.07 ± 0.53; + TGF-β1 THA, 0.62 ± 0.20) (Fig. 3G). However, the mean values of the non-stimulated and stimulated spheroids were not significantly different (−TGF-β1, *p* = 0.068; + TGF-β1, *p* = 0.115). Alcian blue stainings of spheroid section indicated the formation of chondrogenic matrix following induction of chondrogenic differentiation of BM-MSCs from both groups (Fig. 3H). Quantification of the total protein content of chondrogenic spheroids indicated higher protein content of spheroids based on BM-MSCs from POAs in comparison to spheroids based on BM-MSCs from THA (mean protein content per spheroid [µg] ± SD: −TGF-β1 PAO, 28.6 ± 12.7; −TGF-β1 THA, 10.9 ± 6.9; + TGF-β1 PAO, 38.0 ± 14.9; + TGF-β1 THA, 22.9 ± 7.6) (Fig. 3I). The mean values of the non-stimulated spheroids were found to be significantly different (−TGF-β1, *p* = 0.025) and the mean values of the stimulated spheroids were found to be not significantly different (+TGF-β1, *p* = 0.070). The median of the proteoglycan content of the chondrogenic spheroids based on BM-MSCs from the PAO group was found to be higher than the median of the chondrogenic spheroids based on BM-MSCs from the THA group (total proteoglycan, 1.8-fold; proteoglycan normalized to total protein, 2.0-fold) (Fig. 3J, K). However, the median values of the two groups were not significantly different due to large variation in the proteoglycan content in spheroids based on BM-MSCs from PAO (−TGF-β1, *p* = 0.429; +TGF-β1, *p* = 0.430).

Taken together, the analyses of the trilineage differentiation potential indicate that BM-MSCs from samples obtained during PAO are capable of osteogenic, adipogenic and chondrogenic differentiation. BM-MSCs isolated from bone obtained during PAO and THA did not show significant differences in the osteogenic and adipogenic differentiation potential and higher potential for chondrogenic differentiation when considering the trend toward larger spheroids, with higher total protein and proteoglycan content.

### Secretion of signaling molecules

**Fig. 4:**
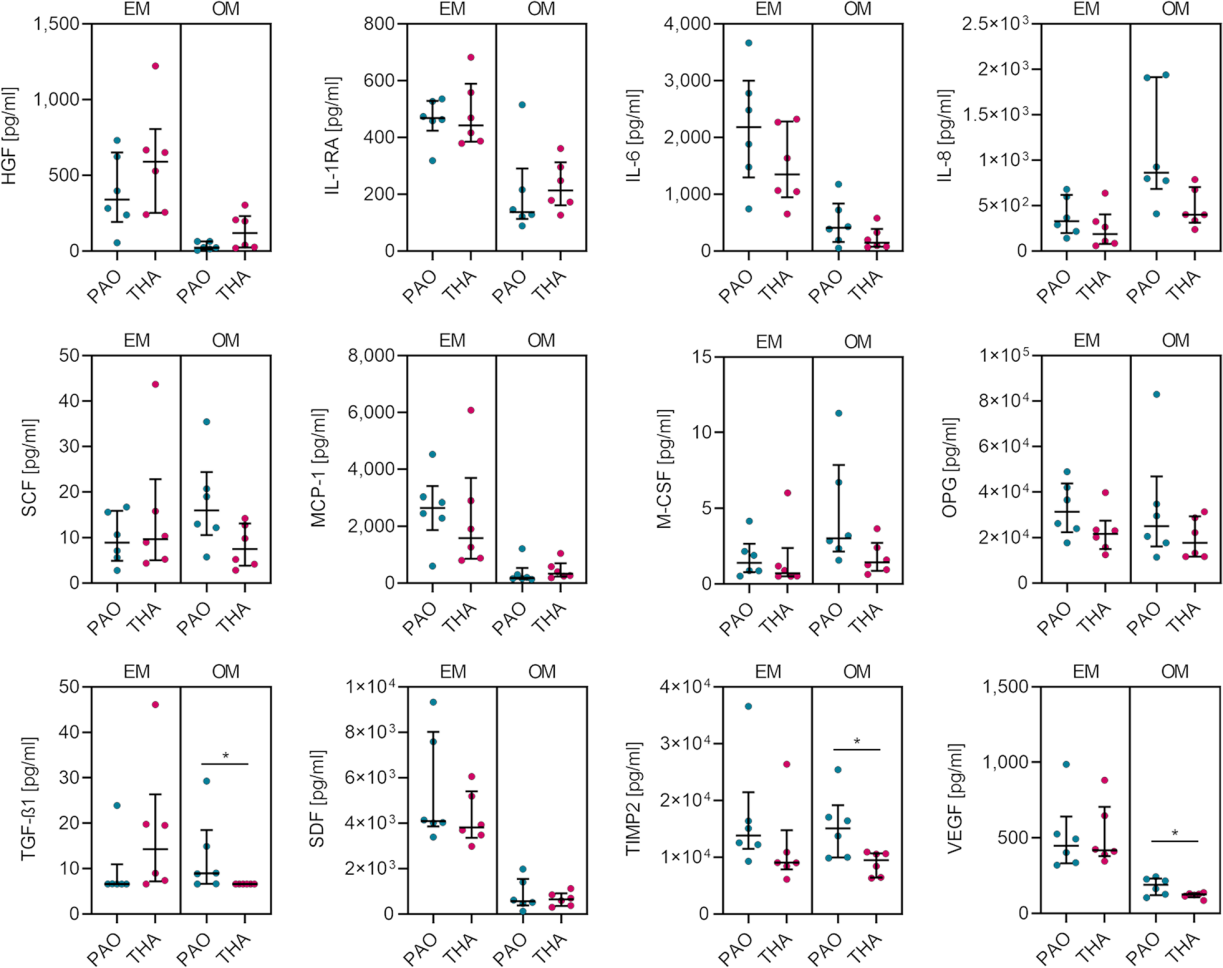
Multiplex analysis of 12 soluble mediators. Cell culture supernatants were collected at day 7 of cell culture in expansion medium (EM) and osteogenic medium (OM). [Median with IQR, levels of significance: *p < 0.05]

In order to evaluate whether the origin of BM-MSCs influences the secretion of signaling molecules under standard and osteogenic culture conditions, a multiplex analysis was performed including 12 cytokines, chemokines, and growth factors that play important roles in either bone metabolism, immunoregulation, and/or matrix remodeling (Fig. 4).

A fold decrease of > 2 was noticed in osteogenically stimulated BM-MSCs compared to non-differentiated BM-MSCs regarding IL-6, MCP-1, HGF, IL-1RA, SDF and VEGF concentrations. IL-8 and M-CSF concentrations were enhanced with a fold increase of > 2 when osteogenic differentiation was induced (only in PAO group). Significantly different concentrations were not observed for the above-mentioned factors between BM-MSCs from PAO and THA in either EM or OM (Additional file 1: Table S3). However, BM-MSCs obtained from PAO secreted significantly more TGF-β1 (1.4-fold, *p* = 0.015) and TIMP2 (1.6-fold, *p* = 0.039) than BM-MSCs from THA following osteogenic stimulation. Although being down-regulated following osteogenic differentiation, BM-MSCs obtained from PAO secreted significantly more VEGF (1.5-fold, *p* = 0.048) than BM-MSCs from THA following osteogenic stimulation.

Overall, BM-MSCs isolated from bone obtained during PAO showed significantly increased TGF-β1, TIMP2, and VEGF levels in cell culture supernatant when cultured under osteogenic conditions.

## Discussion

The aim of this study was to evaluate the potential of bone marrow of periacetabular origin obtained during PAO as a novel source of BM-MSCs. The in-depth analysis and comparison of biological characteristics and functional properties of BM-MSCs isolated from bone obtained during PAO and THA revealed novel information about the influence of donor age on the functionality of these cells.

Overall, patients undergoing PAO were younger compared to patients undergoing THA. Considering this difference, it has to be emphasized that patients scheduled for THA are more likely to suffer from comorbidities like obesity and type-2 diabetes, as these represent major risk factors for the development of osteoarthritis [33, 34]. The majority of PAO patients, on the other hand, is young, healthy and active [35]. Within the framework of the presented study, BM-MNCs were successfully isolated from bone obtained during PAO in 30 of 34 cases. However, the weight of bone marrow contained in the bone samples turned out to be a limiting factor regarding the isolation of BM-MNCs. In the four cases of isolation failure, the sample contained less than 0.24 g bone marrow. Using such small volumes did not result in successful isolation of sufficient amounts of BM-MNCs. Therefore, we recommend considering bone material that grants at least 0.24 g bone marrow to ensure BM-MNC isolation via density gradient centrifugation. Whether other isolation techniques might be more successful in providing sufficiently high cell yields from small bone marrow weights should be further investigated. Comparing bone from THA and PAO, the yield of BM-MNCs relative to the bone marrow mass used, was higher within the THA group. In comparison of using the femoral head after THA and an iliac crest aspiration for the isolation of BM-MNCs in the same patient, Sanchez-Guijo et al., however, did not find such a difference [36]. Considering the respective available literature being very heterogenous, it remains unclear whether the harvesting site is a main factor contributing to significant differences in BM-MNC yield [37–40]. Besides the anatomical site, patient age may be another factor contributing to our results. Andrzejewska et al. investigated the effect of donor age on the content of BM-MNCs isolated from samples obtained during THA [21]. In contrast to our findings, that study did not show a significant influence of advanced donor age on the parameter, although age distribution (average age 38.2 vs. 72.2 years) within groups was comparable to ours. This indicates that the site of sample extraction strongly influences the number of BM-MNCs relative to sample weight. Our determined proportions of BM-MSCs within the BM-MNC population were compatible with reported values from literature [3, 41, 42]. If BM-MNCs were seeded for cell culture, further isolation of BM-MSCs via plastic adherence was possible in every case, in accordance with the ISCT-defined criterion of cell surface marker expression [3]. The potential to obtain BM-MSCs relative to the number of BM-MNCs used was not different at cell culture passage one regarding the two groups. Herrmann et al. reported similar results by comparing bone marrow from the femoral head, the iliac crest and vertebral bodies. A correlation between the yield of BM-MSCs and donor age was not found [43]. In our study, at cell culture passage two, the BM-MSC numbers from PAO samples relative to the numbers of BM-MNCs initially seeded were found to be significantly higher, either with or without correction for the bone marrow weight. This indicates a higher proliferation capacity of BM-MSCs obtained from PAO samples as compared to BM-MSCs obtained from THA samples. The higher proliferation capacity results in an absolute cell number comparable to that of the THA group at cell culture passage two although the bone marrow samples obtained via PAO were significantly smaller. This is in line with the finding of an increased population doubling of BM-MCS from the PAO group as compared to cells from the THA group. These findings are most likely based on the different patient age in both groups since many studies reported decreased proliferation of MSCs from old compared to young donors [22, 44, 45]. Prall et al. investigated the influence of donor age on BM-MSCs from the proximal tibia and iliac crest with regard to cell proliferation and osteogenic differentiation potential, concluding that both harvest site and donor age do not have a significant impact on these cell properties [46]. In view of the heterogeneous findings reported in the literature, it remains uncertain whether the determined difference is based on donor age. In the context of a therapeutic application, however, increased proliferation could be beneficial to achieve higher cell numbers. Overall, the initial cell yield in the PAO group showed a high heterogeneity, which could lead to increased rates of failure with regards to obtaining sufficient cell numbers for clinical applications. However, other applications such as in vitro models and tissue engineering might benefit from an additional source of BM-MSCs from young and healthy donors. This assumption needs to be tested in future studies using larger case numbers to ultimately assess the clinical applicability of early-passage BM-MSCs obtained from PAO. Our data demonstrate that varying patient ages and anatomical sampling positions do not influence the timing of SA-β-gal expression as a common marker for cellular senescence in vitro. SA-β-gal expression, occurs in both groups starting from cell culture passage five. This finding is consistent with previously reported detection of in vitro aging of clinical grade human BM-MSCs [47]. The occurrence of cellular senescence in late cell culture passages involving specific markers for cell cycle arrest (p16, p21) and the DNA damage response pathway (gamma-H2A.X) should be considered in future experimental approaches involving BM-MSCs from PAO samples, as it should be for MSCs from all other cell sources. In the context of cell therapies, the occurrence of cellular senescence may be detrimental depending on the application due to the diminished immunosuppressive and weakened regenerative potential of in vitro ages BM-MCS [48].

Similar to our findings, Sanchez-Guijo et al. did not find significant differences when comparing the multilineage differentiation potential of BM-MSCs obtained from trabecular bone of the femoral head versus from iliac crest bone marrow aspiration [36]. Comparing BM-MSCs isolated from femoral bone fragments and pieces of subchondral bone from the acetabulum, Nguyen et al. reported comparable results with regard to adipogenic differentiation but significantly less calcium deposits following osteogenic differentiation of cells isolated from the subchondral bone of the acetabulum [49]. Given our contrary findings in view of osteogenic differentiation, it is important to clarify that the area where the bone material was taken during PAO does not correspond to the area of subchondral bone used in the above-mentioned study. Regarding the chondrogenic differentiation, BM-MSCs from PAO patients showed a trend of increased spheroid size compared to those from THA patients, regardless of whether they were induced for chondrogenic differentiation. Similar findings were described by Herrmann et al., with larger pellets formed by cells from the iliac crest than from the femoral head [43]. Correlated to the size of the pellets, we found total protein content to be significantly higher in pellets from the PAO group when not stimulated for chondrogenic differentiation. Nevertheless, our study could not determine significant differences in proteoglycan content and proteoglycan proportion within total protein. Therefore, it can be assumed that chondrogenic differentiation did not differ significantly regarding the origin of the bone marrow used to isolate the cells.

BM-MSCs from the PAO group induced for osteogenic differentiation showed increased secretion of TGF-β1, TIMP2, and VEGF compared to cells from the THA group. Besides various functions on a cellular level, TGF-β1 is a key factor in early bone healing and bone homeostasis [50, 51]. Matrix metalloproteases (MMPs) are involved in the degradation and remodeling of extracellular matrix, an important process during endochondral ossification [52]. As important regulatory inhibitors of MMPs, the expression of TIMPs during endochondral ossification is crucial. Not only TGF-β1 and TIMP2 but also VEGF is involved in endochondral ossification, mainly by mediating angiogenesis [53–55]. Generally, local blood supply is one of the most important factors regarding bone healing, and VEGF is the main contributing factor [56]. Overall, the increased secretion of TGF-β1, TIMP2, and VEGF in osteogenically differentiated BM-MSCs from PAO patients compared with those from THA patients may be beneficial for future therapies in the context of bone repair and endochondral ossification. The application of BM-MSCs from PAO samples in the context of cell therapies for bone healing or to stimulate bone formation of impaired periprosthetic bone requires preclinical testing in advanced and multicellular human in vitro models [57–59] and in vivo testing [13].

Our study indicates that isolation of BM-MNCs from PAO samples is possible. The isolated cells are functional in terms of trilineage differentiation and are characterized by higher proliferation capacity if compared to BM-MNCs from THA samples. Due to the focus on a basic characterization of this cell source from young and healthy patients, our feasibility study has limitations in terms of necessary further characterization within the framework of the translational process toward an allogenic cell product. Further characterization of BM-MNCs from PAO samples regarding clonogenicity, chromosomal stability, cell yield, the onset of cell cycle arrest, DNA damage response and the in vitro dysfunctionality following serial passaging, should be performed to ultimately assess the clinical feasibility of this novel cell source.

## Conclusions

Bone obtained during PAO can be a novel source for BM-MSCs. The yield of BM-MSCs from PAO samples relative to the number of BM-MNCs seeded, the weight corrected cell yield and the proliferation capacity were higher as compared to BM-MSCs from THA samples. However, the small sample weight of PAO samples leads to a lower absolute cell yield at cell culture passage one. The absolute number of BM-MSC from PAO samples at cell culture passage two is comparable to that of BM-MSCs from THA samples although characterized by a greater individual variability pointing toward a less stable cell supply of early-passage BM-MSCs. Future experiments need to determine the interplay of donor age, proliferation rate and final yield of BM-MSCs from PAO samples. SA-β-gal expression of BM-MNCs of both groups becomes evident in cell culture passage five. BM-MSCs from the PAO group show trilineage differentiation potential not different from BM-MCSs from the THA group. Upon osteogenic differentiation, BM-MSCs from the PAO group showed increased secretion of TGF-β1, TIMP2, and VEGF, all contributing to endochondral ossification. We propose that bone harvested in the standard course of PAO is a novel reliable source of BM-MSCs from young, healthy donors. With regard to future regenerative therapies and basic musculoskeletal research, we suggest a promising new source for BM-MSCs.

### Supplementary Information

**Additional file 1**. Supplementary tables and figure.

## Data Availability

All data of this study are available from the corresponding author upon reasonable request.

## Abbreviations

ALP: Alkaline phosphatase
BM-MNC: Bone marrow-derived mononuclear cell
BM-MSC: Bone marrow-derived mesenchymal stromal cell
CFU: Colony-forming unit
DMEM: Dulbecco's modified eagle's medium
EM: Expansion medium
FA: Formaldehyde
FACS: Fluorescence activated cell sorting
FBS: Fetal bovine serum
HAS: Human serum albumin
HGF: Hepatocyte growth factor
IL-1RA: Interleukin-1 receptor antagonist
IL-6: Interleukin-6
IL-8: Interleukin-8
IQR: Interquartile range
ISCT: International Society for Cellular Therapy
MCP-1: Monocyte chemoattractant protein-1
M-CSF: Macrophage colony-stimulating factor
MMP: Matrix metalloproteases
MSC: Mesenchymal stromal cell
OM: Osteogenic medium
OPG: Osteoprotegerin
PAO: Periacetabular osteotomy
PBS: Phosphate-buffered saline
pNP: 4-Nitrophenol
pNPP: Para-Nitrophenylphosphate
P1NP: Procollagen type I N-terminal propeptide
SA-β-gal: Senescence-associated beta galactosidase
SCF: Stem cell factor
SD: Standard deviation
SDF: Stromal cell-derived factor
TGF-β1: Transforming growth factor-beta 1
THA: Total hip arthroplasty
TIMP2: Tissue inhibitor of metalloproteinases 2
VEGF: Vascular endothelial growth factor

## References

[1] Ferrin I, Beloqui I, Zabaleta L, Salcedo JM, Trigueros C, Martin AG (2017). Isolation, culture, and expansion of mesenchymal stem cells. Methods Mol Biol.

[2] Jovic D, Yu Y, Wang D, Wang K, Li H, Xu F (2022). A brief overview of global trends in MSC-based cell therapy. Stem Cell Rev Rep.

[3] Dominici M, Le Blanc K, Mueller I, Slaper-Cortenbach I, Marini F, Krause D (2006). Minimal criteria for defining multipotent mesenchymal stromal cells. the International society for cellular therapy position statement. Cytotherapy..

[4] Shang F, Yu Y, Liu S, Ming L, Zhang Y, Zhou Z (2021). Advancing application of mesenchymal stem cell-based bone tissue regeneration. Bioact Mater.

[5] Sarugaser R, Hanoun L, Keating A, Stanford WL, Davies JE (2009). Human mesenchymal stem cells self-renew and differentiate according to a deterministic hierarchy. PLoS ONE.

[6] Killington K, Mafi R, Mafi P, Khan WS (2018). A systematic review of clinical studies investigating mesenchymal stem cells for fracture non-union and bone defects. Curr Stem Cell Res Ther.

[7] Freitas J, Santos SG, Goncalves RM, Teixeira JH, Barbosa MA, Almeida MI (2019). Genetically engineered-MSC therapies for non-unions, delayed unions and critical-size bone defects. Int J Mol Sci.

[8] Gomez-Barrena E, Padilla-Eguiluz N, Rosset P, Gebhard F, Hernigou P, Baldini N (2020). Early efficacy evaluation of mesenchymal stromal cells (MSC) combined to biomaterials to treat long bone non-unions. Injury.

[9] Wildemann B, Ignatius A, Leung F, Taitsman LA, Smith RM, Pesantez R (2021). Non-union bone fractures. Nat Rev Dis Primers.

[10] Bao C, He C (2021). The role and therapeutic potential of MSC-derived exosomes in osteoarthritis. Arch Biochem Biophys.

[11] Zhao X, Zhao Y, Sun X, Xing Y, Wang X, Yang Q (2020). Immunomodulation of MSCs and MSC-derived extracellular vesicles in osteoarthritis. Front Bioeng Biotechnol.

[12] Xiang XN, Zhu SY, He HC, Yu X, Xu Y, He CQ (2022). Mesenchymal stromal cell-based therapy for cartilage regeneration in knee osteoarthritis. Stem Cell Res Ther.

[13] Hochmann S, Ou K, Poupardin R, Mittermeir M, Textor M, Ali S (2023). The enhancer landscape predetermines the skeletal regeneration capacity of stromal cells. Sci Transl Med.

[14] Brown C, McKee C, Bakshi S, Walker K, Hakman E, Halassy S (2019). Mesenchymal stem cells: cell therapy and regeneration potential. J Tissue Eng Regen Med.

[15] Seetharaman R, Mahmood A, Kshatriya P, Patel D, Srivastava A (2019). An overview on stem cells in tissue regeneration. Curr Pharm Des.

[16] Moll G, Ankrum JA, Kamhieh-Milz J, Bieback K, Ringden O, Volk HD (2019). Intravascular mesenchymal stromal/stem cell therapy product diversification: time for new clinical guidelines. Trends Mol Med.

[17] Pierini M, Di Bella C, Dozza B, Frisoni T, Martella E, Bellotti C (2013). The posterior iliac crest outperforms the anterior iliac crest when obtaining mesenchymal stem cells from bone marrow. J Bone Joint Surg Am.

[18] Li J, Wong WH, Chan S, Chim JC, Cheung KM, Lee TL (2011). Factors affecting mesenchymal stromal cells yield from bone marrow aspiration. Chin J Cancer Res.

[19] Ogawa T, Sugaya H, Hara Y, Yoshii Y, Ochiai N, Yamazaki M (2021). Does the radius contain bone marrow mesenchymal stem cells?-A comparison between cells of the iliac crest and radius in Kienbock's disease. J Hand Surg Asian Pac.

[20] Drela K, Stanaszek L, Snioch K, Kuczynska Z, Wrobel M, Sarzynska S (2020). Bone marrow-derived from the human femoral shaft as a new source of mesenchymal stem/stromal cells: an alternative cell material for banking and clinical transplantation. Stem Cell Res Ther.

[21] Andrzejewska A, Catar R, Schoon J, Qazi TH, Sass FA, Jacobi D (2019). Multi-parameter analysis of biobanked human bone marrow stromal cells shows little influence for donor age and mild comorbidities on phenotypic and functional properties. Front Immunol.

[22] Carvalho MS, Alves L, Bogalho I, Cabral JMS, da Silva CL (2021). Impact of donor age on the osteogenic supportive capacity of mesenchymal stromal cell-derived extracellular matrix. Front Cell Dev Biol.

[23] Katz JN, Arant KR, Loeser RF (2021). Diagnosis and treatment of hip and knee osteoarthritis: a review. JAMA.

[24] Mareschi K, Ferrero I, Rustichelli D, Aschero S, Gammaitoni L, Aglietta M (2006). Expansion of mesenchymal stem cells isolated from pediatric and adult donor bone marrow. J Cell Biochem.

[25] Ganz R, Klaue K, Vinh TS, Mast JW (1988). A new periacetabular osteotomy for the treatment of hip dysplasias. Technique and preliminary results. Clin Orthop Relat Res.

[26] Wassilew GI, Hofer A, Rakow A, Gebhardt S, Hoffmann M, Janz V (2022). Minimally invasive periacetabular osteotomy for adult hip dysplasia. Oper Orthop Traumatol.

[27] Lochel J, Janz V, Perka C, Hofer A, Zimmerer A, Wassilew GI (2021). A new rectus and Sartorius sparing approach for Periacetabular osteotomy in patients with developmental dysplasia of the Hip. J Clin Med.

[28] Fischer M, Schoon J, Freund E, Miebach L, Weltmann KD, Bekeschus S (2022). Biocompatible gas plasma treatment affects secretion profiles but not osteogenic differentiation in patient-derived mesenchymal stromal cells. Int J Mol Sci.

[29] Wick MR (2019). The hematoxylin and eosin stain in anatomic pathology-An often-neglected focus of quality assurance in the laboratory. Semin Diagn Pathol.

[30] Ode A, Schoon J, Kurtz A, Gaetjen M, Ode JE, Geissler S (2013). CD73/5'-ecto-nucleotidase acts as a regulatory factor in osteo-/chondrogenic differentiation of mechanically stimulated mesenchymal stromal cells. Eur Cell Mater.

[31] Rakow A, Schoon J, Dienelt A, John T, Textor M, Duda G (2016). Influence of particulate and dissociated metal-on-metal hip endoprosthesis wear on mesenchymal stromal cells in vivo and in vitro. Biomaterials.

[32] Davis LA, Dienelt A, Zur Nieden NI (2011). Absorption-based assays for the analysis of osteogenic and chondrogenic yield. Methods Mol Biol..

[33] Swain S, Sarmanova A, Coupland C, Doherty M, Zhang W (2020). Comorbidities in osteoarthritis: a systematic review and meta-analysis of observational studies. Arthritis Care Res (Hoboken).

[34] Duclos M (2016). Osteoarthritis, obesity and type 2 diabetes: the weight of waist circumference. Ann Phys Rehabil Med.

[35] Lorenzon P, Scalvi A, Scalco E (2020). The painful hip in young adults between impingement and mild dysplasia: clinical and instrumental diagnostical criteria. Acta Biomed..

[36] Sanchez-Guijo FM, Blanco JF, Cruz G, Muntion S, Gomez M, Carrancio S (2009). Multiparametric comparison of mesenchymal stromal cells obtained from trabecular bone by using a novel isolation method with those obtained by iliac crest aspiration from the same subjects. Cell Tissue Res.

[37] Cavallo C, Boffa A, de Girolamo L, Merli G, Kon E, Cattini L (2022). Bone marrow aspirate concentrate quality is affected by age and harvest site. Knee Surg Sports Traumatol Arthrosc..

[38] Narbona-Carceles J, Vaquero J, Suarez-Sancho S, Forriol F, Fernandez-Santos ME (2014). Bone marrow mesenchymal stem cell aspirates from alternative sources: is the knee as good as the iliac crest?. Injury.

[39] Fragkakis EM, El-Jawhari JJ, Dunsmuir RA, Millner PA, Rao AS, Henshaw KT (2018). Vertebral body versus iliac crest bone marrow as a source of multipotential stromal cells: comparison of processing techniques, tri-lineage differentiation and application on a scaffold for spine fusion. PLoS ONE.

[40] Min WK, Bae JS, Park BC, Jeon IH, Jin HK, Son MJ (2010). Proliferation and osteoblastic differentiation of bone marrow stem cells: comparison of vertebral body and iliac crest. Eur Spine J.

[41] Alvarez-Viejo M, Menendez-Menendez Y, Blanco-Gelaz MA, Ferrero-Gutierrez A, Fernandez-Rodriguez MA, Gala J (2013). Quantifying mesenchymal stem cells in the mononuclear cell fraction of bone marrow samples obtained for cell therapy. Transplant Proc.

[42] Schafer R, DeBaun MR, Fleck E, Centeno CJ, Kraft D, Leibacher J (2019). Quantitation of progenitor cell populations and growth factors after bone marrow aspirate concentration. J Transl Med.

[43] Herrmann M, Hildebrand M, Menzel U, Fahy N, Alini M, Lang S (2019). Phenotypic characterization of bone marrow mononuclear cells and derived stromal cell populations from human iliac crest, vertebral body and femoral head. Int J Mol Sci.

[44] Mendes SC, Tibbe JM, Veenhof M, Bakker K, Both S, Platenburg PP (2002). Bone tissue-engineered implants using human bone marrow stromal cells: effect of culture conditions and donor age. Tissue Eng.

[45] Fan M, Chen W, Liu W, Du GQ, Jiang SL, Tian WC (2010). The effect of age on the efficacy of human mesenchymal stem cell transplantation after a myocardial infarction. Rejuvenation Res.

[46] Prall WC, Saller MM, Scheumaier A, Tucholski T, Taha S, Bocker W (2018). Proliferative and osteogenic differentiation capacity of mesenchymal stromal cells: influence of harvesting site and donor age. Injury.

[47] Oja S, Komulainen P, Penttilä A, Nystedt J, Korhonen M (2018). Automated image analysis detects aging in clinical-grade mesenchymal stromal cell cultures. Stem Cell Res Ther.

[48] Zhang Y, Ravikumar M, Ling L, Nurcombe V, Cool SM (2021). Age-related changes in the inflammatory status of human mesenchymal stem cells: implications for cell therapy. Stem Cell Reports.

[49] Nguyen VT, Tessaro I, Marmotti A, Sirtori C, Peretti GM, Mangiavini L (2019). Does the harvesting site influence the osteogenic potential of mesenchymal stem cells?. Stem Cells Int.

[50] Sarahrudi K, Thomas A, Mousavi M, Kaiser G, Kottstorfer J, Kecht M (2011). Elevated transforming growth factor-beta 1 (TGF-beta1) levels in human fracture healing. Injury.

[51] Crane JL, Xian L, Cao X (2016). Role of TGF-beta signaling in coupling bone remodeling. Methods Mol Biol.

[52] Ortega N, Behonick DJ, Werb Z (2004). Matrix remodeling during endochondral ossification. Trends Cell Biol.

[53] Hu K, Olsen BR (2016). The roles of vascular endothelial growth factor in bone repair and regeneration. Bone.

[54] Dai J, Rabie AB (2007). VEGF: an essential mediator of both angiogenesis and endochondral ossification. J Dent Res.

[55] Hu K, Olsen BR (2016). Osteoblast-derived VEGF regulates osteoblast differentiation and bone formation during bone repair. J Clin Invest.

[56] Keramaris NC, Calori GM, Nikolaou VS, Schemitsch EH, Giannoudis PV (2008). Fracture vascularity and bone healing: a systematic review of the role of VEGF. Injury.

[57] Schoon J, Hesse B, Rakow A, Ort MJ, Lagrange A, Jacobi D (2020). Metal-specific biomaterial accumulation in human peri-implant bone and bone marrow. Advanced Science.

[58] Scheinpflug J, Pfeiffenberger M, Damerau A, Schwarz F, Textor M, Lang A (2018). Journey into bone models: a review. Genes.

[59] Scheinpflug J, Höfer CT, Schmerbeck SS, Steinfath M, Doka J, Tesfahunegn YA (2023). A microphysiological system for studying human bone biology under simultaneous control of oxygen tension and mechanical loading. Lab Chip.

