# Supplementary material for: Bone marrow from periacetabular osteotomies as a novel source for human mesenchymal stromal cells
Source: Stem Cell Res Ther. 2023 Nov 3;14:315. doi: 10.1186/s13287-023-03552-9 (PMC10625289; doi:10.1186/s13287-023-03552-9)
Supplement: Supplementary file 1 — Additional file 1. Supplementary tables and figure. [file 13287_2023_3552_MOESM1_ESM.docx]

**ADDITIONAL FILE 1**

**Titel**

Bone marrow from periacetabular osteotomies as a novel source for human mesenchymal stromal cells

**Authors**

*Maximilian Handke, Anastasia Rakow, Debora Singer, Lea Miebach, Frank Schulze, Sander Bekeschus, Janosch Schoon, Georgi I. Wassilew*

__________________________________________________________________________

**Table S1** Patient and sample data

| donor# | age | sex | surgical treatment | BM weight [g] | BM-MNC isolation | number of BM-MNCs isolated [x 10^6^] | BM-MNC-FACS | H&E  histology | CFU | MSC-FACS | post-thaw viability | viability | cell number | β-gal | Alizarin Red | ALP activity | P1NP assay | NileRed/ DAPI | total protein | proteo-glycan | spheroid histology |
| --- | --- | --- | --- | --- | --- | --- | --- | --- | --- | --- | --- | --- | --- | --- | --- | --- | --- | --- | --- | --- | --- |
| 1 | 18 | f | PAO | 1.41 | x | 32.8 |  |  |  | x |  | x | x |  |  | x | x | x | x | x | x |
| 2 | 29 | m | PAO | 1.11 | x | 20.3 |  |  |  | x |  | x | x |  | x | x | x | x | x | x | x |
| 3 | 31 | f | PAO | 0.57 | x | 21.2 |  |  |  | x | x | x | x | x | x | x | x | x | x | x | x |
| 4 | 36 | f | PAO | 0.45 | x | 11.8 |  |  |  | x |  | x | x |  | x | x | x | x | x | x | x |
| 5 | 25 | m | PAO | 0.68 | x | 28.3 |  |  |  | x | x | x | x | x | x | x | x | x | x | x | x |
| 6 | 23 | f | PAO | 0.78 | x | 40.5 |  |  |  | x | x | x | x | x | x | x | x | x | x | x | x |
| 7 | 34 | f | PAO | n.d. |  | n.d. |  | x |  |  |  |  |  |  |  |  |  |  |  |  |  |
| 8 | 29 | f | PAO | n.d. |  | n.d. |  | x |  |  |  |  |  |  |  |  |  |  |  |  |  |
| 9 | 27 | m | PAO | n.d. |  | n.d. |  | x |  |  |  |  |  |  |  |  |  |  |  |  |  |
| 10 | 25 | f | PAO | n.d. |  | n.d. |  | x |  |  |  |  |  |  |  |  |  |  |  |  |  |
| 11 | 33 | f | PAO | n.d. |  | n.d. |  | x |  |  |  |  |  |  |  |  |  |  |  |  |  |
| 12 | 40 | f | PAO | 0.51 | x | 38.5 | x |  |  |  |  |  |  |  |  |  |  |  |  |  |  |
| 13 | 32 | m | PAO | 0.72 | x | 12.9 | x | x |  |  |  |  |  |  |  |  |  |  |  |  |  |
| 14 | 27 | m | PAO | 0.23 | x | 21.8 | x |  |  |  |  |  |  |  |  |  |  |  |  |  |  |
| 15 | 39 | m | PAO | 0.71 | x | 20.6 | x |  |  |  |  |  |  |  |  |  |  |  |  |  |  |
| 16 | 33 | f | PAO | 0.29 | x | 9.3 | x |  |  |  |  |  |  |  |  |  |  |  |  |  |  |
| 17 | 38 | f | PAO | 0.58 | x | 59.2 | x |  |  |  |  |  |  |  |  |  |  |  |  |  |  |
| 18 | 36 | f | PAO | 0.36 | x | 4.8 |  |  |  |  |  |  |  |  |  |  |  |  |  |  |  |
| 19 | 39 | m | PAO | 0.56 | x | 21.8 |  |  |  |  |  |  |  |  |  |  |  |  |  |  |  |
| 20 | 29 | m | PAO | 0.24 | x | 4.7 |  |  |  |  | x |  |  | x |  |  |  |  |  |  |  |
| 21 | 30 | m | PAO | 0.60 | x | 8.2 |  |  |  |  |  |  |  |  |  |  |  |  |  |  |  |
| 22 | 35 | m | PAO | 0.44 | x | 7.9 |  |  |  |  | x |  |  | x |  |  |  |  |  |  |  |
| 23 | 44 | f | PAO | 0.49 | x | 10.0 |  |  |  |  |  |  |  |  |  |  |  |  |  |  |  |
| 24 | 42 | f | PAO | 0.25 | x | 2.1 |  |  |  |  |  |  |  |  |  |  |  |  |  |  |  |
| 25 | 31 | f | PAO | 0.35 | x | 6.8 |  |  |  |  |  |  |  |  |  |  |  |  |  |  |  |
| 26 | 36 | f | PAO | 0.27 | x | 3.0 |  |  |  |  |  |  |  |  |  |  |  |  |  |  |  |
| 27 | 30 | f | PAO | 0.47 | x | 20.3 |  |  |  |  | x |  |  | x |  |  |  |  |  |  |  |
| 28 | 43 | m | PAO | n.d. | x | 23.1 |  |  | x |  |  |  |  |  |  |  |  |  |  |  |  |
| 29 | 40 | f | PAO | n.d. | x | 23.4 |  |  | x |  |  |  |  |  |  |  |  |  |  |  |  |
| 30 | 37 | m | PAO | n.d. | x | 29.7 |  |  | x |  |  |  |  |  |  |  |  |  |  |  |  |
| 31 | 41 | m | PAO | n.d. | x | 32.8 |  |  | x |  |  |  |  |  |  |  |  |  |  |  |  |
| 32 | 74 | f | THA | 4.44 | x | 505.0 | x |  |  | x | x | x | x | x |  | x | x | x | x | x | x |
| 33 | 81 | f | THA | 2.57 | x | 180.5 | x |  |  | x | x | x | x | x | x | x | x | x |  |  |  |
| 34 | 58 | m | THA | 4.53 | x | 235.5 | x |  |  | x | x | x | x | x | x | x | x | x | x | x | x |
| 35 | 86 | f | THA | 4.41 | x | 247.5 | x |  |  | x |  | x | x |  | x | x | x | x | x | x | x |
| 36 | 62 | m | THA | 3.89 | x | 236.5 | x |  |  | x | x | x | x | x | x | x | x | x | x | x | x |
| 37 | 59 | m | THA | 3.14 | x | 403.0 | x |  |  | x | x | x | x | x | x | x | x | x | x | x | x |
| 38 | 83 | f | THA | n.d. |  | n.d. |  | x |  |  |  |  |  |  |  |  |  |  |  |  |  |
| 39 | 63 | m | THA | n.d. | x | 54.5 |  |  | x |  |  |  |  |  |  |  |  |  |  |  |  |
| 40 | 41 | f | THA | n.d. | x | 118.2 |  |  | x |  |  |  |  |  |  |  |  |  |  |  |  |
| 41 | 65 | f | THA | n.d. | x | 94.8 |  |  | x |  |  |  |  |  |  |  |  |  |  |  |  |
| 42 | 61 | f | THA | n.d. | x | 40.2 |  |  | x |  |  |  |  |  |  |  |  |  |  |  |  |

Abbreviations: ALP, alkaline phosphatase; β-gal, β-galactosidase staining; BM, bone marrow; BM-MNCs, bone marrow mononuclear cells; CFU, colony forming unit assay; FACS, fluorescence activated cell sorting; H&E, haematoxylin & eosin; MSC, mesenchymal stem/stromal cell; n.d., not determined; P1NP, human procollagen type I N-terminal propeptide; PAO, periacetabular osteotomy; THA, total hip arthroplasty

**Table S2** Preoperative medication and concomitant diseases of patients whose bone marrow was used for BM-MSC isolation and subsequent BM-MSC characterization.

| donor# | age | sex | surgical treatment | preoperative concomitant medication | comorbidities |
| --- | --- | --- | --- | --- | --- |
| 1 | 18 | f | PAO | none | irritable bowel syndrome, migraine, atopic dermatitis, allergic rhinitis, eating disorder, depressive disorder |
| 2 | 29 | m | PAO | none | history of multiple fractures and surgeries of the right-sided lower leg, spinal disc herniation L4/5 |
| 3 | 31 | f | PAO | none | none |
| 4 | 36 | f | PAO | none | history of right-sided humerus fracture, history of appendectomy |
| 5 | 25 | m | PAO | none | history of appendectomy |
| 6 | 23 | f | PAO | L-thyroxine, venlafaxine | hypothyroidism, depressive disorder, nephrolithiasis, history of surgical treatment of spinal disc herniation L4/5 |
| 32 | 74 | f | THA | candesartan, lercanidipine, allopurinol | arterial hypertension, hyperuricemia, history of lumbar spinal disc herniation, history of SARS-CoV2-infection |
| 33 | 81 | f | THA | ASA, atorvastatin, lisinopril, metoprolol, lercanidipine, indapamide, metformin, insulin glulisine, insulin glargine | arterial hypertension, hyperlipoproteinemia, peripheral arterial disease, IDDM, diabetic polyneuropathy, obesity |
| 34 | 58 | m | THA | none | none |
| 35 | 86 | f | THA | ASA, candesartan, amlodipine, atorvastatin, vitamin B12, macrogol | arterial hypertension. coronary artery disease, hyperuricemia, varicosis, unilateral carotid artery stenosis, history of right-sided total knee arthroplasty, left-sided total hip arthroplasty, cholecystectomy and hysterectomy |
| 36 | 62 | m | THA | spironolactone, simvastastin, bisoprolol | arterial hypertension, proteinuria, primary hyperaldosteronism / Conn's syndrome, history of surgery for pectus excavatum |
| 37 | 59 | m | THA | hydrochlorothiazide, spironolactone, ramipril / amlodipine, carvedilol, metformin, sitagliptin | arterial hypertension, NIDDM, COPD, obesity |

Abbreviations: ASA, acetylsalicylic acid; COPD, chronic obstructive pulmonary disease; IDDM, insulin dependent diabetes mellitus; NIDDM, non-insulin-dependent diabetes mellitus; PAO, periacetabular osteotomy; THA, total hip arthroplasty

**Figure S1**

**
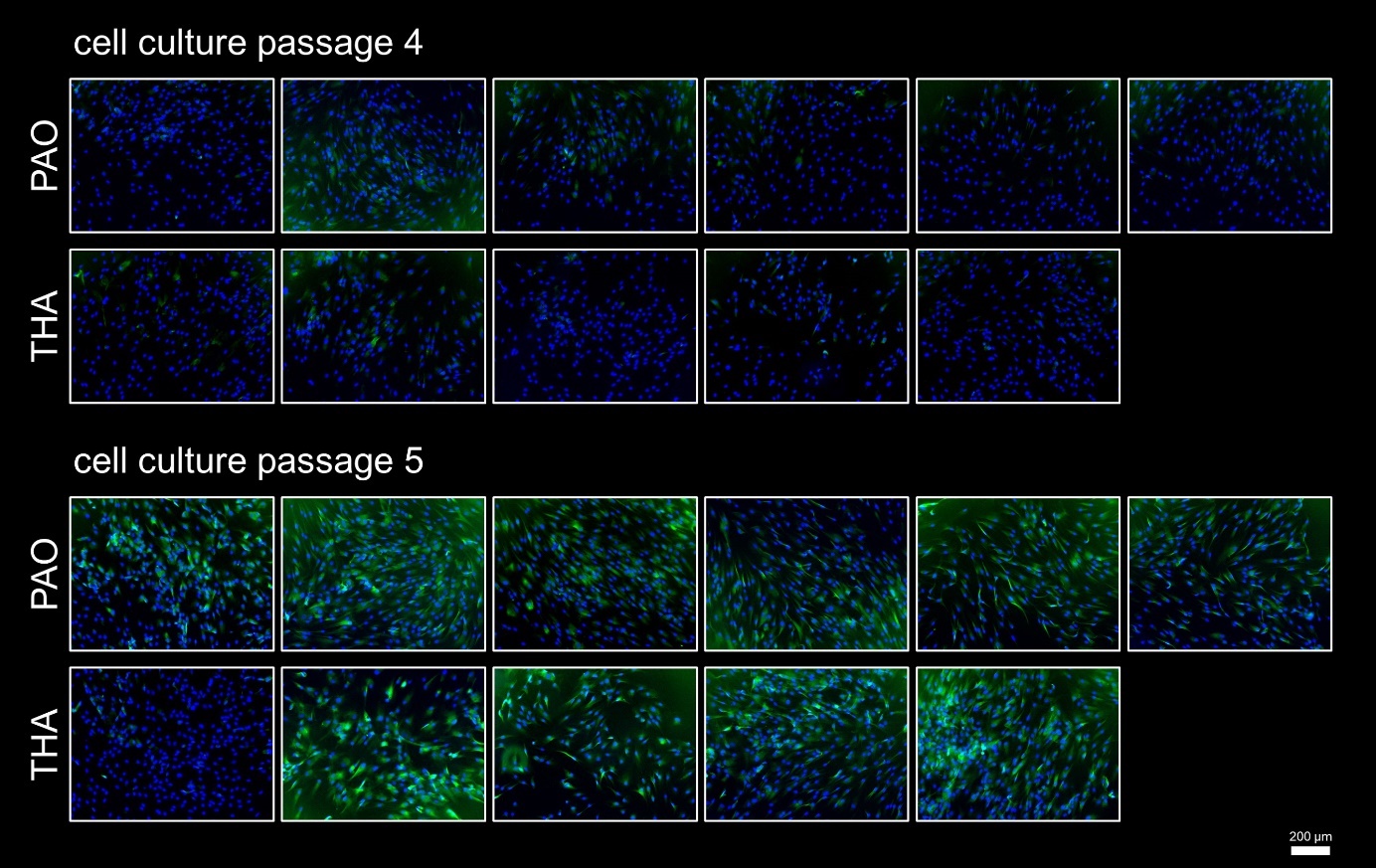
**

**Fig. S1** Fluorescence images of BM-MSCs from cell culture passage four and five isolated from six PAO samples and five THA samples stained for senescence-associated beta galactosidase. Green, beta galactosidase; blue, nuclei.

**Table S3** Median values of multiplexed cell signaling molecules and *p*-values of the according group comparisons.

| analyte | PAO - EM  median [pg/ml] | THA - EM  median [pg/ml] | *p*-value | PAO - OM  median [pg/ml] | PAO - OM  median [pg/ml] | *p*-value |
| --- | --- | --- | --- | --- | --- | --- |
| HGF | 339 | 589 | *0.276* | 22.3 | 118 | *0.096* |
| IL-1RA | 469 | 443 | *0.747* | 137 | 213 | *0.310* |
| IL-6 | 2,182 | 1,350 | *0.214* | 407 | 146 | *0.175* |
| IL-8 | 328 | 186 | *0.306* | 861 | 400 | *0.055* |
| SCF | 8.88 | 9.65 | *0.937* | 16.0 | 7.50 | *0.066* |
| MCP-1 | 2,642 | 1,584 | *0.751* | 175 | 330 | *0.240* |
| M-CSF | 1.39 | 0.71 | *0.455* | 3.02 | 1.43 | *0.116* |
| OPG | 31,374 | 21,609 | *0.134* | 25,060 | 17,696 | *0.485* |
| TGF-ß1 | 6.61 | 14.3 | *0.113* | 8.94 | 6.61 | ***0.015*** |
| SDF | 4,084 | 3,811 | *0.310* | 562 | 642 | *0.542* |
| TIMP2 | 13,811 | 9,046 | *0.065* | 15,070 | 9,492 | ***0.039*** |
| VEGF | 448 | 418 | *0.818* | 189 | 125 | ***0.048*** |

Abbreviations: EM, expansion medium, OM, osteogenic medium
